# Transcriptome and metabolome insights into closely related upland cotton (*Gossypium hirsutum*) genotypes during differing responses to progressive soil drying

**DOI:** 10.1186/s12870-026-08125-5

**Published:** 2026-01-29

**Authors:** Annelie Marquardt, Philippe Moncuquet, Katrina J Broughton, Robert E Sharwood, Warren C Conaty

**Affiliations:** 1 Agriculture and Food, CSIRO, St Lucia, QLD Australia; 2 Agriculture and Food, CSIRO, Black Mountain, ACT Australia; 3 Agriculture and Food, CSIRO, Narrabri, NSW Australia; 4 Hawkesbury Institute for the Environment, Western Sydney University, Richmond, NSW Australia

**Keywords:** Cotton, Drought, Rainfed, Fraction of transpirable soil water, FTSW, Soil drying, Transcriptome, Metabolome

## Abstract

**Background:**

Cotton is an important crop worldwide for producing natural fibres for textile, among other uses. Water input for cotton production is tightly linked to yield and ongoing climate changes require purpose-bred cotton genotypes able to be grown productively in water-conservative environments, particularly as most fertile land must be prioritised for food production. Here we investigate metabolome and transcriptome changes in the leaves of two closely related upland cotton (*Gossypium hirsutum*) genotypes with a differing fraction of transpirable soil water (FTSW) threshold trait, undergoing a progressive soil drying treatment in a controlled environment.

**Results:**

We show that gene expression and metabolite changes are present earlier in the genotype with a higher FTSW threshold (early-saver; ES), matching physiology data collected at the same time. There were unique gene expression patterns present in the ES genotype involving upregulation of predicted MEX1 and pGlct-2 genes involved in starch breakdown, as well as lower transcript levels of a predicted cold and drought responsive CORA-like protein. Integration of metabolome and transcriptome showed associations between genes and metabolites in each genotype, notable in ES were multiple aquaporin genes with L-proline, suggesting these may be key players in the higher FTSW-threshold response of cotton to progressive soil-drying.

**Conclusions:**

This work contributes to the understanding of genes and metabolites involved in cotton’s response to progressive soil drying and highlights potential differences between high and low FTSW threshold phenotypes. This will provide a basis for further studies to pursue the breeding of cotton genotypes with improved productivity and adaptation to water limited and rainfed production systems.

**Supplementary Information:**

The online version contains supplementary material available at 10.1186/s12870-026-08125-5.

## Background

Cotton is a globally important crop producing natural fibres with a wide range of applications from textile production to cattle feed. The predominantly planted cotton species is upland cotton (*Gossypium hirsutum*) and is cultivated in more than 80 countries around the world.

Reduced soil water availability is a major contributor to yield losses in cropping systems worldwide. In Australia, water availability stands out as the most critical abiotic factor limiting cotton production [1–3]. The challenges posed by water limitation are being intensified by human-induced climate change and a rapidly growing global population. Forecasts for the coming century predict increased variability in rainfall, more frequent droughts, and rising global temperatures, all of which threaten the availability and reliability of water resources for irrigated agriculture [4]. Water shortages are further compounded by rising competition for water between agricultural, urban, and environmental needs. Although the Australian cotton industry has historically achieved high yields through irrigation, reduced water supplies are prompting a shift toward partially irrigated and dryland farming systems. As a result, breeding cotton varieties that can maintain high yields under water-limited conditions is essential. This effort relies on identifying water-saving traits in existing cotton genotypes to support the development of drought-tolerant cultivars.

One such water-saving trait is the ability to reduce transpiration rates as soil moisture declines. A common pattern observed across some species and environments is that transpiration remains steady until soil moisture drops below a certain threshold, triggering stomatal closure and a subsequent linear decline in transpiration [5, 6]. Soil water content may be quantified as the fraction of transpirable soil water (FTSW), a concept introduced by Ritchie et al. [7]. The FTSW threshold—where transpiration begins to decline—shows considerable genotypic variation in several crops [6, 8–10]. For instance, Devi et al. [6] shows FTSW thresholds in peanut genotypes range from 0.22 to 0.71, Shekoofa et al. [11] shows soybean ranges from 0.18 to 0.8, and Gholipoor et al. [12] that maize hybrids vary from 0.33 to 0.60. Similar variability has been documented in sorghum [13], field pea [14], potato [8], and turfgrass [15]. In upland cotton (*G. hirsutum*), recent studies have reported FTSW thresholds between 0.29 and 0.39 in four U.S. cultivars [16], and between 0.13 and 0.29 in six genotypes—five from Australia and one from India [17].

Genotypes that begin conserving water at higher FTSW values can preserve soil moisture earlier in the season, making more water available during later drought periods. This strategy supports sustained productivity under prolonged water stress, in contrast to genotypes with lower FTSW thresholds that exhaust water resources more quickly [13, 18]. The superior performance of these water-conserving genotypes under limited water conditions makes them valuable candidates for breeding programs aimed at improving yields in partially irrigated and dryland systems. Linking these phenotypic traits to genetic markers could enable more precise and efficient breeding strategies.

The FTSW threshold trait is most likely a complex, quantitative trait. Although methods such as genome-wide association studies (GWAS) have proven to be useful for such traits, the approach is impractical for finding genetic regions of interest linked to high or low FTSW threshold due to the time-consuming and laborious nature of phenotyping. Therefore, an approach aimed to further understanding of the molecular mechanisms of FTSW threshold variability presents an alternative option. This may be achieved through investigation of gene expression and metabolism changes that occur in genotypes which differ in their FTSW threshold when soil moisture declines. Such work may lead to a greater understanding of the mechanism of how cotton responds to soil drying as well as isolating genes that may behave differently between cotton genotypes.

Transcriptome studies, specifically RNA-seq, has been used to study the molecular underpinnings of drought responses in countless species to find gene networks of interest. Typically, findings include changes in genes involved in photosynthesis, hormone signalling, amino acid metabolism, secondary metabolite production, fatty acid and carbohydrate metabolism (1; and references therein). As gene expression data in the form of RNA-seq represents a point in time of transient transcript abundance and is not necessarily linked to protein abundance or activity upon metabolites, is it informative to include other omics information when studying complex plant responses such as to drought. Metabolome studies provide data of the end products of cellular processes and therefore, combined with transcriptome data, allow a more comprehensive understanding of connections between gene expression patterns and corresponding metabolic changes occurring.

This study was undertaken to find transcriptomic and metabolomic differences between two closely related *G. hirsutum* genotypes (CSIRO breeding lines) when exposed to a progressive soil drying treatment in a glasshouse setting. The genotypes display differing phenotypes in FTSW threshold during declining plant available water, wherein genotype CSX2027 (referred to as early-saver; ES) decreases its stomatal conductance at a higher FTSW than genotype CSX8521 (referred to as late-saver; LS) [17]. This ES phenotype is more suited to productivity under water limited environments by exploiting a more conservative water use pattern, allowing soil water to be available for use later in the crop’s lifecycle. RNA-seq was used to generate transcriptome data, and targeted and untargeted approaches were used to generate metabolomics data to match physiological measurements [17] over three timepoints of a progressive soil drying treatment. We hypothesised that (a) gene and metabolite profiles would reflect changes in leaf level physiology (gas exchange, photosynthetic rate), with similar regulatory pathways invoked for both genotypes, and (b) the timing of when these responses occur would reflect the genotype’s differing FTSW threshold, suggesting that it is largely the sensing of soil moisture that triggers a drought response of the plant rather than a difference in the way the plants respond. The aim of this work is to build an understanding of the genes and metabolites involved in the regulation of physiological processes during progressive soil drying in closely related genotypes with differing FTSW thresholds. This work is important as it will provide a basis for targeting breeding cotton genotypes with improved productivity and adaptation to water limited and rainfed production systems.

## Methods

### Plant growth

A glasshouse experiment was conducted to study two closely related *G. hirsutum* genotypes which differed in their response to progressive soil drying. Gas exchange measurements were taken with matching leaf samples for transcriptome and metabolome analyses.

### Plant material

Breeding lines CSX2027 and CSX8521 from CSIRO, Australia were selected based on results of previous studies which highlighted differences in transpiration responses to progressive soil drying. CSX2027 had displayed reduced transpiration at a higher level of soil moisture remaining (higher fraction of transpirable soil water (FTSW) threshold) than CSX8521 (lower FTSW threshold) in a previous study [17]. In this study, CSX2027 was referred to as early-saver (ES) and CSX8521 was referred to as late-saver (LS), to describe their water saving patterns when experiencing progressive soil drying.

### Glasshouse experiment

The experiment took place from November to December 2020 in Narrabri, NSW in Australia (-30.207, 149.597) in glasshouse facilities with natural light conditions (14 h d^− 1^ of PPFD > 2,000 mmol m^− 2^ s^− 1^). Full details can be found in previous work [17], and a brief description provided here.

### Soil composition

Plants were grown in a mixture of 3:1 field soil: perlite, in replicates per genotype. Field soil was obtained from local fields and is classified as a Grey Vertosol (Australian soil classification) with pH 8.0-8.8 and clay fraction percentage of 60–65% and low organic matter content [19]. This was augmented by adding 10 g of MULTIgrow^®^ basal fertiliser (13.1% N, 4.5% P, 7.2% K, 15.4% S and 2.4% Ca) (Incitec Pivot Fertilisers, Melbourne, Australia) to the soil surface of each plot and was dissolved with hand-held irrigation. Before treatment began all soils were kept well-watered with hand held watering or daily irrigation via drip irrigators to saucers underneath pots.

### Progressive soil drying treatment

Uniformity of plant growth before the beginning of the treatment was ensured by sowing eight plants per pot and then thinning to one plant per pot for the beginning of the treatment. Just prior to first square (23 days after planting (DAP)), plants were blocked by size and divided into two water treatments; well-watered (WW; control) and a progressive water deficit (WD). At 26 DAP, all pots were watered to field capacity. The surface of each pot was covered with aluminium foil to prevent soil evaporation before the pots were weighed to determine the initial pot weight. Well-watered plants were watered each morning (~ 0800 h) to the initial pot weight recorded on the first day of weighing. Water deficit plants were exposed to progressive soil drying (reduction in the FTSW) by imposing a daily water deficit of 65% of the previous day’s water use. This enabled extension of the drying cycle over several weeks to more accurately represent field drying conditions. The weight of each pot was recorded each morning and the amount of water transpired per day was calculated as the change in pot weight between successive days.

### Fraction of transpirable soil water (FTSW)

The total FTSW was calculated as the difference between the initial and final pot weight. The FTSW on each day was calculated using the formula:

$$Fraction transpirable soil water \left(FTSW\right)=\:\frac{(Daily\:pot\:weight-Final\:pot\:weight)}{(Initial\:pot\:weight-Final\:pot\:weight)}$$

### Sample preparation

For four replicates of each treatment (WW and WD), at four timepoints, one leaf sample was processed (64 samples in total). These timepoints were T0; 26 DAP and before progressive soil drying treatment began with transpiration ratio of 1, T1; 36 DAP and 10d after treatment began with a transpiration ratio of approximately 0.7, T2; 40 DAP and 14d after treatment began with a transpiration ratio of approximately 0.4, and T3; 47 DAP and 21d after treatment began with a transpiration ratio of approximately 0.1 [17].

### Transcriptome preparation

#### RNA extraction

Leaf samples were stored at -80 °C preceding grinding to powder with liquid N-cooled canisters in a Retsch Ball Mill (Retsch GmbH, Haan, Germany) at 30.0 Hz for 35 s. From this leaf powder, 50–100 mg was used for RNA extraction. RNA extraction was performed with Qiagen RNeasy Plant Mini Kit (Qiagen, Hilden, Germany) following manufacturer’s instructions using RLT buffer, with the addition of dithiothreitol (0.3% w/v; Promega, Madison, USA), proteinase K (0.33% w/v, Sigma-Aldrich, St Louis, MO, USA) and polyvinylpyrrolidone-40 (PVP-40; 2% w/v; Sigma-Aldrich St Louis, MO, USA). Additionally, DNase treatment with on-column RNase-free DNase kit (Qiagen, Hilden, Germany) was performed. Amount and quality of RNA was checked on NanoDrop (NanoDrop One, ThermoFisher Scientific, Madison, USA) and through 1.5% TBE gel electrophoresis.

#### RNA sequencing and processing

Total RNA from 64 leaves were submitted to GENEWIZ (Azenta, China) for poly-A selection, non-strand specific library preparation and sequencing using Illumina NovaSeq to generate approximately 20 M 150 bp paired-end reads. Reads were trimmed and quality-checked with fastp v22.0 [20] with default setting. Alignment to reference genome was performed using STAR v2.7.9a [21] with settings of --outFilterScoreMinOverLread 0.4 --outFilterMatchNminOverLread 0.4. Alignment reports collated with Multiqc v1.11 [22] and python v3.9.4. Leaf samples yielded on average 24.7 million uniquely mapped reads, with an average mapping rate of 89.7%. Read counts were obtained with htseq-count [23] with python v3.9.4.

### Transcriptome reference genome

Reference genome used was *Gossypium hirsutum* (AD1) ‘TM-1’ genome UTX v2.1 [24], obtained from CottonGen.org and indexed using STAR v2.7.9a [21] (for alignment).

### Transcriptome analysis

Hierarchical clustering and principal component analysis were performed using R v4.0.5 [25].

Differential expression calculation performed using DESeq2 v1.30.1 [26] in R v4.0.5 [25] and transcripts with adjusted p-value of < 0.01 were considered differentially expression genes (DEGs) where the FDR method applied to correct p-values was Benjamini-Hochberg for differential expression between firstly four replicates of WW (control) and four replicates of WD samples for each timepoint and genotype, and secondly, between four replicates of ES genotype and LS genotype (control) at each timepoint and treatment.

Over-representation analysis of differentially expressed genes in GO categories and KEGG pathways was performed using R package gprofiler2 v0.2.2 [27] in R v4.0.5 [25] using *Arabidopsis thaliana* database, ‘fdr’ correction method in an ordered analysis with custom background (*A. thaliana* gene annotation matches (obtained from *G. hirsutum* UTXv2.1 reference genome [24] annotation files at Cottongen.org) to the full list of reference genome genes present).

UpSet plots were generated using UpSetR package v1.4.0 [28] in R v4.0.5 [25] and Venn diagrams generated using online tool from Bioinformatics Gent at https://bioinformatics.psb.ugent.be/webtools/Venn/.

### Metabolome preparation

#### Sample preparation

Briefly, 10.0 mg of freeze dried plant material was reconstituted with 100 µL MilliQ Water and combined with 450 µL of ice-cold (-20 °C) methanol: ethanol (50% v/v; LiChrosolv^®^, Merck, Darmstadt, Germany) spiked with 1 ppm Succinic Acid 13C2 (Cambridge Scientific) and vortexed for 2 min. The samples were centrifuged (Centrifuge 5430R, Eppendorf, Hamburg, Germany) at 14,000 rcf at 4 °C for 5 min. The supernatant was transferred and filtered using a positive pressure manifold (Agilent PPM48 Processor, Agilent Technologies, Santa Clara, California, USA) with Captiva EMR cartridges (40 mg, 1 mL; Agilent, Mulgrave, VIC, Australia) to remove the lipid fraction. The cartridges were then washed with two 200 µL aliquots of MilliQ: methanol: ethanol (50/25/25% v/v/v). The combined filtered supernatant and cartridge washes, representing the polar metabolite fraction, were combined in a 1.5 mL high recovery vial (30 µL reservoir, silanized glass vials, Agilent Technologies, Mulgrave, Australia) and dried in a SpeedVAC (10 mBar). The polar metabolite fraction was reconstituted with 100 µL MilliQ: methanol (80/20% v/v) comprised 100 ppb of L-Phenylalanine (1–13 C).

Central carbon metabolism metabolites were measured on an Agilent Infinity Flex II UHPLC coupled to an Agilent 6470 Triple Quadrupole Mass Spectrometer (QqQ-MS) following [29]. Untargeted polar metabolites were analysed using an Agilent 6546 liquid chromatography time-of-flight mass spectrometer (LC-QToF-MS), as per [30]. Samples were first blank subtracted, normalised to the spiked internal standard (Succininc Acid 13C2) and sample biomass (10.0 mg).

#### Data processing

Missing values were replaced by 1/5 of min positive values of their corresponding variables. The acquired data were then normalised by the sample median, log transformed (base 10) and scaled using the mean-centred value and divided by the standard deviation of each variable.

### Metabolome analysis

Data were subjected to univariate statistical analysis and over-representation analysis (fisher’s exact test) using MetaboAnalystR 4.0 [31] in R v4.0.5 [25] with the KEGG pathway database of *Arabidopsis thaliana*.

Principal Component Analysis (PCA) and Partial Least Squares Discriminant Analysis (PLS-DA) were performed using mixOmics v6.26.0 [32] in R v4.0.5 [25]. For the PLS-DA, samples of the timepoint T0 designated as the WD treatment were reclassified as WW (as T0 samples were taken preceding onset of the water-deficit treatment). Variable Importance in projection (VIP) scores from the PLS-DA were cut off at VIP > 1.

### Integrated transcriptome and metabolome analysis

Integration of transcriptome and metabolome was performed using PLS2/sPLS in the R package mixOmics v6.26.0 [32] in R v4.0.5 [25]. Only data from T0, T1, T2 and T3 of the WD treated plants was used (where T0 samples were taken preceding the water-deficit treatment). The transcriptome was input in the form of count normalised DESeq2 values [26, 33] filtered by mean absolute deviation (MAD) of greater than 0.65 to retain approximately 8,000 genes. Metabolome data was input as normalised data from the data processing step. Mode = ‘canonical’ was used with metabolome as the independent variable (X) and transcriptome as dependent (Y). Tuning was performed to determine two components would be retained in the analysis, with emphasis on the first component. Final run was performed with five folds and nrepeat = 100.

Integration was performed with the research question of ‘which features (genes and metabolites) have the greatest covariance during the progressive soil drying treatment for each genotype’. This was achieved by structuring the analysis to find the genes and metabolites that were perturbed together over the course of the soil drying for each genotype and comparing these between ES and LS.

## Results

**Fig. 1:**
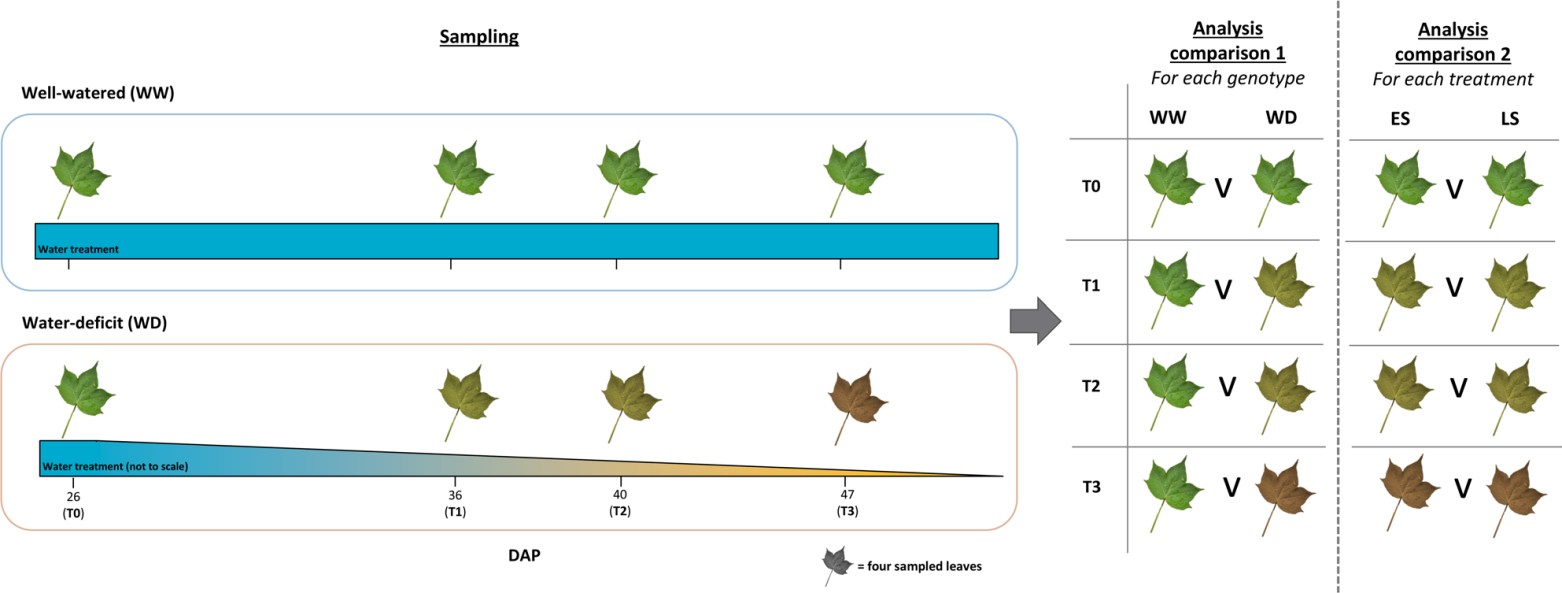
Diagram of glasshouse progressive soil drying experiment sample collection and comparison for differential expression/abundance analysis. This produced differentially expressed genes (DEGs) and differentially abundance metabolites (DAMs) due to water-deficit (WD) treatment, between control (WW) and WD samples, as well as between genotypes early-saver (ES) and late-saver (LS)

**Fig. 2:**
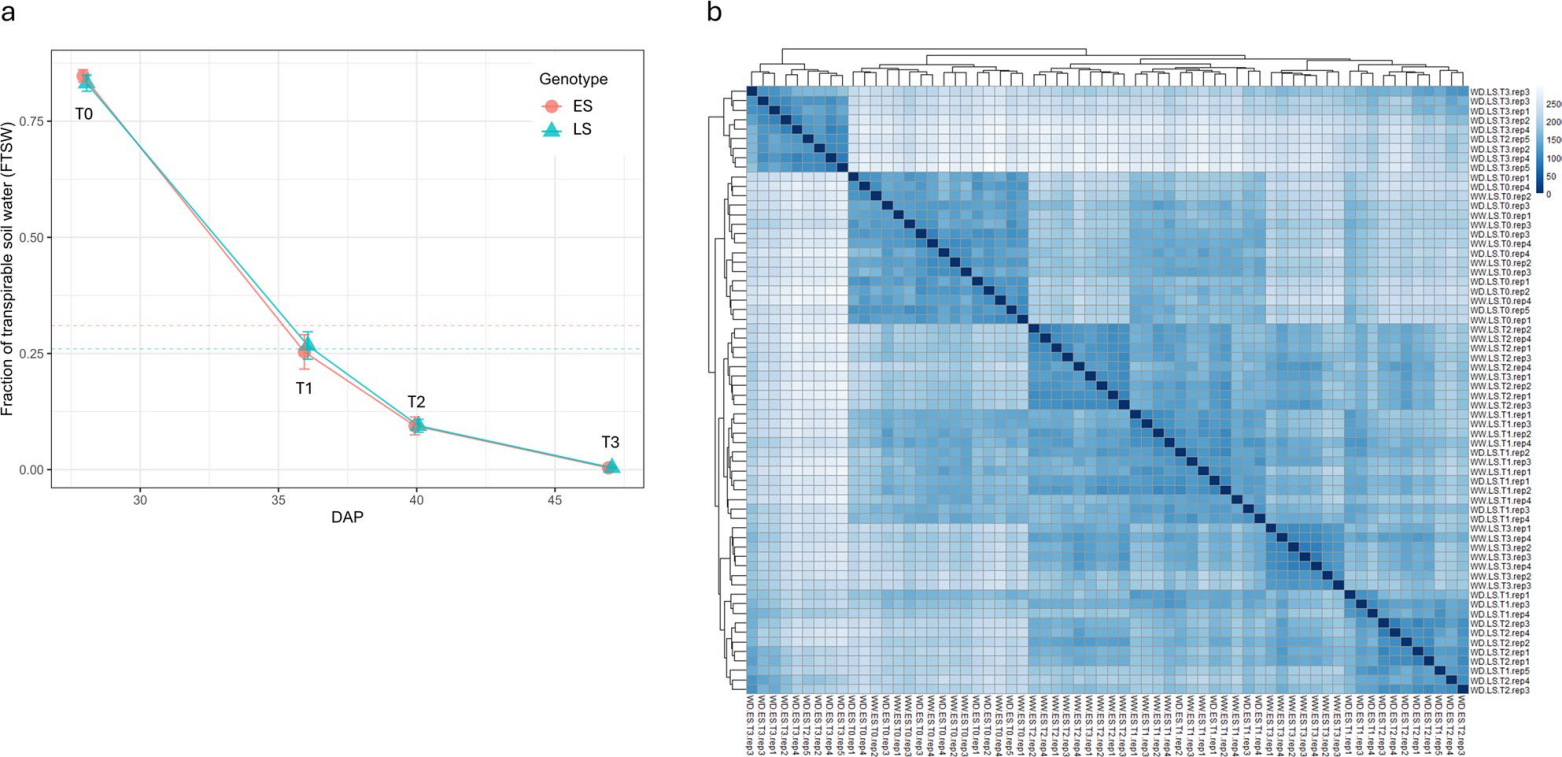
Sample collection timepoint and correlation of samples. **a** Fraction of transpirable soil water (FTSW) value at sampling of T0, T1, T2 and T3 timepoints and corresponding days after planting (DAP) showing dashed line of approximate FTSW threshold of early-saver (ES) and late-saver (LS) genotypes. At T1, the ES genotype had passed its FTSW threshold, by T2 the LS genotype had also passed its threshold. **b** Sample correlation of global expression profiles of the 64 samples in the transcriptome using Euclidean distance, showing clustering of samples

Two cotton genotypes were studied for their difference in fraction of transpirable soil water (FTSW) threshold, wherein CSX2027 (early-saver; ES) limits transpiration at a higher FTSW than CSX8521 (late-saver; LS), enabling it to conserve water in the soil profile for later-use in the season. Leaf tissue for metabolome and transcriptome data generation was collected from both genotypes at four timepoints with differing water-deprivation states (T0, T1, T2 and T3) combined with physiology data in the form of gas exchange measurements. Noting T0 sampling timepoint was collected preceding water-deficit treatment, being a secondary control. Diagram of sampling can be seen in Fig. 1. For the water-deficit treated plants, the approximate FTSW values at each collection timepoint were 100% at T0 (secondary control), 25% at T1, 15% at T2 and 1% at T3 (Fig. 2a). The FTSW threshold of ES is approx. 31%, while that of LS is approx. 25% [17].

Physiology data showed the ES genotype had significantly decreased transpiration rate, photosynthetic rate and stomatal conductance at T1 (approx. 25% FTSW), while LS showed a slight, non-significant decrease. Both genotypes had substantially decreased all three parameters by the T2 (approx. 15% FTSW) timepoint and further so by T3 (approx. 1% FTSW).

### Transcriptomic characteristics of progressive soil-drying treatment

To understand the effect of the progressive soil-drying treatment on the cellular activity of both genotypes, transcriptomics in the form of poly(A) RNA-sequencing (RNA-seq) was undertaken. An average of 90% of reads mapped to the reference genome across samples. More than 83% of genes present in the reference genome were expressed in both genotypes across all samples. Biological replicates of samples were assessed for quality with a correlation analysis. All four biological replicates of each treatment and timepoint combination clustered together in the correlation analysis indicating sample quality was satisfactory (Fig. 2b). The T0 samples (secondary control) clustered together showing the similarity of both genotype’s samples before the water-deficit treatment was imposed. Both genotypes often clustered together for each treatment/timepoint combination revealing their responses were likely to be similar.

**Fig. 3:**
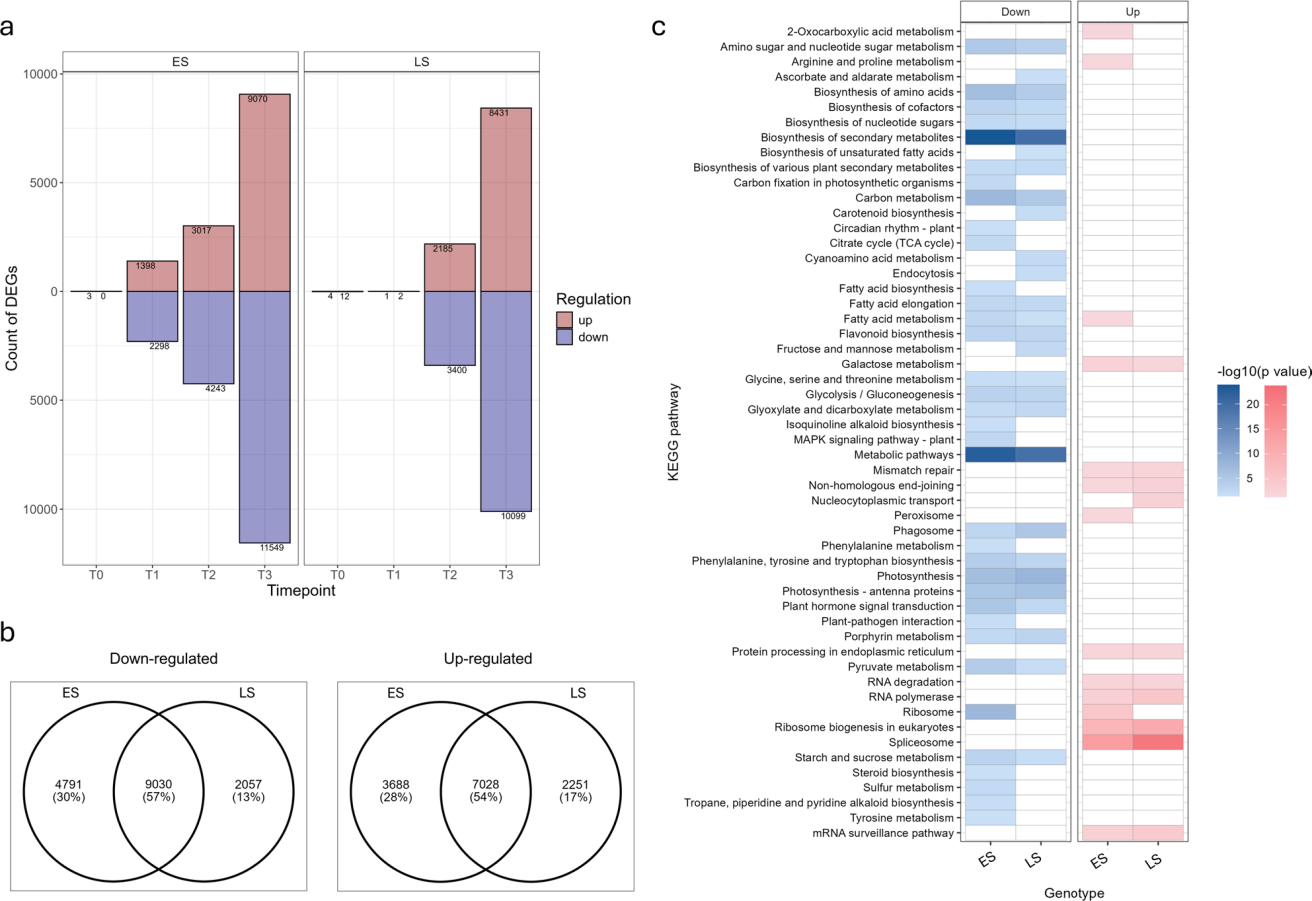
Comparison of up-regulated and down-regulated genes in early-saver (ES) and late-saver (LS) genotypes differing in their sensitivity to soil drying. **a** Graph of differentially expressed gene (DEG) counts in ES and LS genotypes showing the number of those that were up- and downregulated at the four timepoints of sample collection. ES shows a substantial number of DEGs at T1, which LS does not. **b** Overlap of genes significantly up- and down-regulated throughout the progressive soil drying treatment between ES and LS genotypes. **c** KEGG Pathways flagged as of interest in over representation analysis (ORA) on Arabidopsis match to DEGs from ES and LS genotypes. Heatmap colours represent log10 scaled p values for each KEGG pathway ORA result

Differential expression analysis was performed between controls (well-watered; WW) and progressive-soil drying treatment plants (water-deficit; WD) within each timepoint (Fig. 1). In the ES genotype at T1, a total number of 3,696 differentially expressed genes (DEGs) were identified between WW and WD plants. At the same timepoint only three were found in LS. This was consistent with ES having reached its FTSW threshold at T1, while LS had not. At T2, ES had 7,260 DEGs while LS had 5,585, suggesting LS had also reached its FTSW threshold. At T3, both genotypes had substantial DEG counts (20,619 in ES and 18,530 in LS) consistent with severe water stress, which was observed in physiology data. It was noted there were consistently more downregulated genes than upregulated at each timepoint where the FTSW threshold had been reached (Fig. 3a). The full list of DEGs can be found in Additional File 1. The commonality of DEGs between genotypes can be seen in Venn diagrams in Fig. 3b where the majority of DEGs were common to both genotypes (57% of upregulated genes and 54% of downregulated).

DEGs present in all three timepoints where the FTSW threshold was reached (i.e. consistently differentially expressed) in ES (T1, T2 and T3) revealed 554 genes consistently upregulated and 902 genes consistently downregulated. While in LS (T2 and T3), 1338 genes were consistently upregulated and 2420 downregulated. Of these, 376 were commonly upregulated and 718 commonly downregulated between the two genotypes which reflected the consistent and common gene expression changes in the response to soil-drying. These common gene expression changes were investigated by GO and KEGG pathway over-representation analyses (ORA) (Fig. 3c). Common upregulated DEGs were enriched (fdr-corrected pval < 0.01) in processes including ‘response to heat’, ‘response to temperature stimulus’, ‘protein folding’ and ‘response to abiotic stress’ and KEGG pathway ‘protein processing in endoplasmic reticulum’. While common downregulated genes were enriched for photosynthesis related genes, particularly in ‘light harvesting’ and ‘light reactions’, ‘chlorophyll biosynthesis’, ‘porphyrin metabolism, as well as ‘pentose phosphate cycle’ and ‘photosynthesis dark reactions’, further KEGG pathways which were not directly photosynthesis-related were ‘glyoxylate and dicarboxylate metabolism, ‘biosynthesis of secondary metabolites as well as ‘ascorbate and aldarate metabolism’. These were considered typical in leaves responding to a water-deprived state. The full list can be found in Additional File 2.

### Differences between ES and LS gene expression during progressive soil-drying

Around 30% of all up- or downregulated DEGs were unique to ES (3,688 and 4,791, respectively), and around 15% were unique to LS (2,251 and 2,057, respectively) (Fig. 3b).

The DEGs that were uniquely downregulated in ES were enriched in the KEGG pathways including ‘ribosome’, ‘biosynthesis of secondary metabolites’, ‘amino sugar and nucleotide metabolism’, ‘plant hormone transduction’ and others, notably also including the ‘MAPK signalling pathway’. There were no KEGG pathways enriched in the uniquely downregulated DEGs in LS, and one GO term: ‘biological process of cytoskeleton organization’. Those uniquely upregulated in ES were enriched in KEGG pathways ‘ribosome’, ‘spliceosome’ and ‘RNA degradation’ as well as many GO term biological processes including ‘translation’, ‘protein metabolic process’, ‘response to abiotic stimulus’, including ‘ncRNA metabolic process’ as well as others. While in LS four KEGG pathways were enriched: ‘spliceosome’, ‘nucleocytoplasmic transport’, ‘nucleotide excision repair’, and ‘ribosome biogenesis’. For full list of terms/pathways enriched uniquely in ES and LS see Additional File 3. Many GO term biological process were enriched in LS, similar to ES. Including many RNA metabolism process child terms and ‘ncRNA metabolic process’.

To investigate further, those genes that were consistently differentially expressed (present at each timepoint after FTSW threshold had been reached) that were unique to each genotype were found using UpSet/Venn diagrams [28]. Unique to ES were 14 upregulated and 16 downregulated DEGs found consistently in T1, T2 and T3 (Table 1), while 86 upregulated and 127 downregulated DEGs were only present in LS. Lists of these genes are provided in Additional File 4. They included genes of transcription factors such as MYB44 and ERFs (upregulated in LS only), MYB30 (downregulated in LS only), predicted myb-related protein 305 and WRKY transcription factor 54 (downregulated in ES only), and predicted homeobox-leucine zipper protein HAT5-like (upregulated in ES only), and were found in both the A and D cotton genomes. The genes uniquely upregulated in ES from T1 (considered an early and consistent response) included *A05G048700* a BURP domain RD22-like precursor, *A11G226000* a predicted maltose excess protein 1 (MEX1), and *D13G201800* a predicted plastidic glucose transporter 2 (pGlct-2), the latter two of which are involved in starch metabolism, specifically starch breakdown.

**Table 1:** Genes differentially expressed in early-saver (ES) genotype during progressive soil-drying treatment (T1, T2 and T3) and not in late-saver (LS) genotype at any timepoint, indicating genes which May be linked to a unique response of the ES genotype. Genes from both A and D cotton genomes represented

Regulation	Gene	Annotation (NCBI)	Arabidopsis match
Up	*A03G028100*	PREDICTED: probable N-acetyltransferase HLS1-like [*Gossypium hirsutum*]	AT2G30090.1
	*A05G048700*	BURP domain protein RD22-like precursor [*Gossypium arboreum*]	AT5G25610.1
	*A05G059000*	PREDICTED: CBL-interacting serine/threonine-protein kinase 5-like [*Gossypium hirsutum*]	AT5G10930.1
	*A05G242400*	PREDICTED: RNA-binding protein 2 [*Gossypium arboreum*]	AT1G21320.2
	*A06G032640*	PREDICTED: WD and tetratricopeptide repeats protein 1-like [*Gossypium arboretum*]	AT5G10940.2
	*A07G135100*	PREDICTED: CDPK-related kinase 3-like isoform X1 [*Gossypium hirsutum*]	AT2G46700.1
	*A11G124100*	PREDICTED: uncharacterized protein LOC107924407 [*Gossypium hirsutum*]	AT4G02715.2
	*A11G174800*	PREDICTED: chaperone protein dnaJ 11, chloroplastic-like [*Gossypium hirsutum*]	AT2G17880.1
	*A11G226000*	PREDICTED: maltose excess protein 1, chloroplastic-like [*Gossypium hirsutum*]	AT5G17520.1
	*A12G175190*	PREDICTED: homeobox-leucine zipper protein HAT5-like isoform X1 [*Gossypium arboretum*]	AT3G01470.1
	*D02G088700*	hypothetical protein GOBAR_DD00025 [*Gossypium barbadense*]	AT1G27460.1
	*D08G270400*	hypothetical protein GOBAR_AA39728 [*Gossypium barbadense*]	AT2G19810.1
	*D09G057401*	PREDICTED: uncharacterized protein LOC105798727 isoform X1 [*Gossypium raimondii*]	AT3G03790.1
	*D13G201800*	PREDICTED: probable plastidic glucose transporter 2 isoform X1 [*Gossypium hirsutum*]	AT1G67300.3
Down	*A01G052200*	PREDICTED: LEAF RUST 10 DISEASE-RESISTANCE LOCUS RECEPTOR-LIKE PROTEIN KINASE-like 2.1 [*Gossypium hirsutum*]	AT5G38260.1
	*A06G187200*	PREDICTED: cytochrome c oxidase subunit 5b-1, mitochondrial [*Gossypium hirsutum*]	AT1G80230.1
	*A07G038850*	PREDICTED: protein CHUP1, chloroplastic-like [*Gossypium arboreum*]	AT3G25690.6
	*A07G140600*	PREDICTED: delta(8)-fatty-acid desaturase 2-like [*Gossypium hirsutum*]	AT2G46210.1
	*A08G085200*	PREDICTED: mitochondrial outer membrane protein porin 4-like [*Gossypium hirsutum*]	AT5G57490.1
	*A09G027100*	PREDICTED: myb-related protein 305-like [*Gossypium hirsutum*]	AT5G40350.1
	*A09G154600*	PREDICTED: aspartic proteinase PCS1-like [*Gossypium hirsutum*]	AT5G02190.1
	*A11G177300*	PREDICTED: probable LRR receptor-like serine/threonine-protein kinase At4g36180 [*Gossypium hirsutum*]	AT4G36180.1
	*D05G264400*	PREDICTED: probable isoaspartyl peptidase/L-asparaginase 2 [*Gossypium raimondii*]	AT3G16150.1
	*D05G305200*	PREDICTED: phosphate transporter PHO1 homolog 3-like [*Gossypium hirsutum*]	AT1G14040.1
	*D08G049700*	hypothetical protein POPTR_002G190000v3 [*Populus trichocarpa*]	AT2G47110.2
	*D08G110708*	hypothetical protein B456_004G118400, partial [*Gossypium raimondii*]	AT4G20850.1
	*D08G110716*	PREDICTED: tripeptidyl-peptidase 2-like isoform X1 [*Gossypium hirsutum*]	AT4G20850.1
	*D08G173800*	PREDICTED: extra-large guanine nucleotide-binding protein 3-like isoform X1 [*Gossypium hirsutum*]	AT1G31930.6
	*D09G212700*	PREDICTED: uncharacterized protein LOC107928502 [*Gossypium hirsutum*]	AT3G59780.1
	*D13G007900*	probable WRKY transcription factor 54 [*Gossypium hirsutum*]	AT2G40750.1

**Fig. 4:**
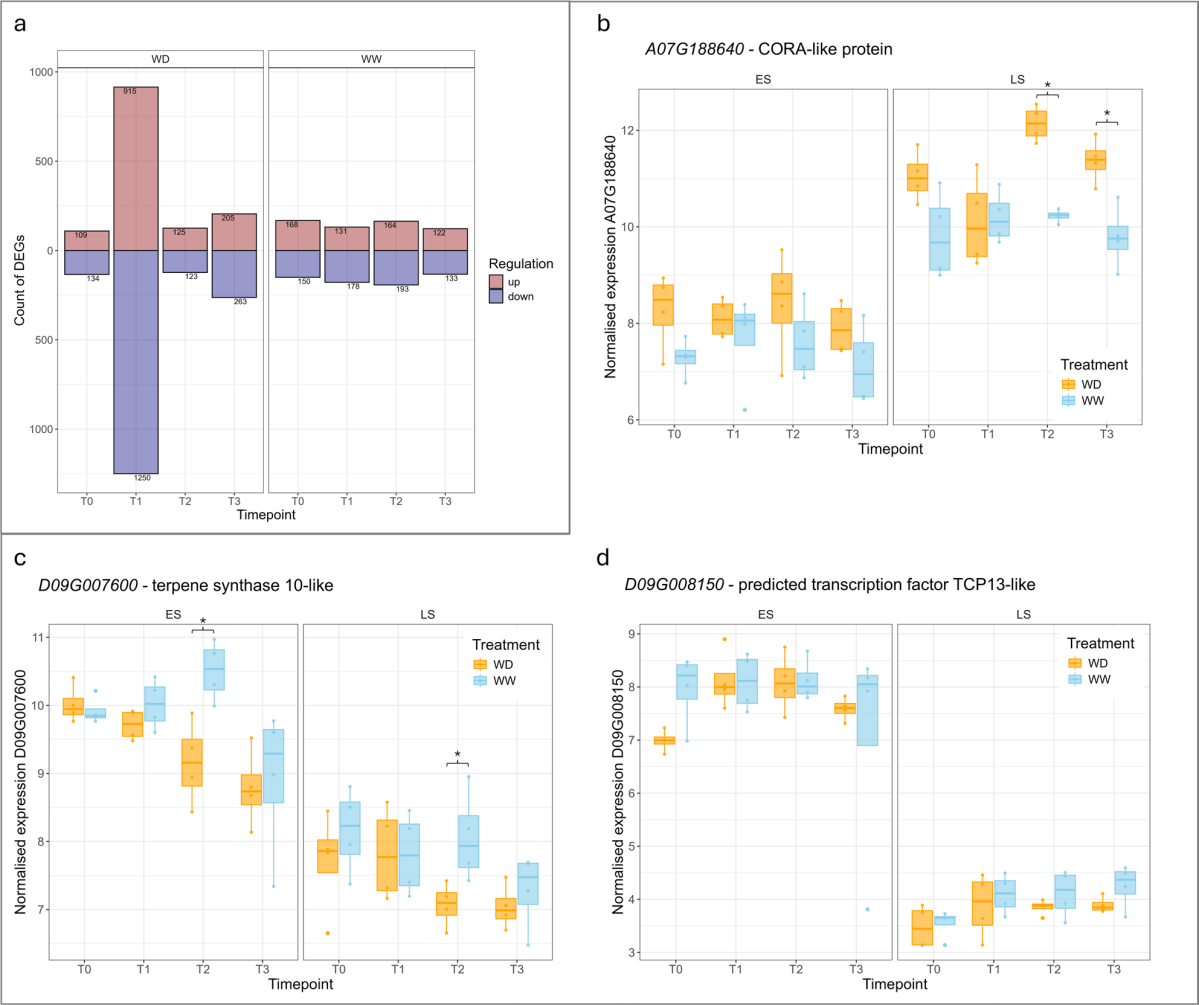
Comparison of up-regulated and down-regulated genes between early-saver (ES) and late-saver (LS; ‘control’) genotypes differing in their sensitivity to soil drying. **a** graph of differentially expressed gene (DEG) counts between WD leaves for each genotype, and WW leaves for each genotype. Showing the number of those that were up- and downregulated in each water-treatment for ES compared to LS (control) at the four timepoints. A similar number of DEGs were consistently present between the two genotypes with the exception of T1 in the WD treated plants. **b-d** relative expression levels of genes that were consistently differentially expressed by ES compared to LS within each timepoint and water treatment (adjusted *p*-value < 0.01). Shows normalised expression levels for each genotype and water treatment across the four sampling timepoints (T0 is secondary control). The gene for Cora-like protein (b) was of particular interest due to showing responsiveness to soil drying in LS (T2 and T3) but not in ES. Asterix above the WD and WW boxplots in b-d indicates a statistically significant difference between the WW and WD treatments (adjusted *p*-value < 0.01)

In addition, differential expression analyses were also performed between genotypes at each timepoint for both WW and WD samples (Figs. 1 and 4a), in this case LS was considered the ‘control’ in the comparison. This was to find any consistent DEGs between genotypes, given their closely related pedigree. This also helped to address the issue where the nature of the experiment meant T3 timepoint water-deficit samples were extremely stressed and, therefore, the assumption of the differential expression analysis of DESeq2 that the majority of genes are not differentially expressed was considered with caution (the majority of genes would potentially be differentially expressed in such a severe water-deprived state of T3). The T3 WD samples compared between genotypes were likely to be similar, and therefore may have showed specific differences in gene expression between genotypes in the severely water-deprived state. The results of the analysis showed a reasonably consistent number of DEGs between ES and LS at each timepoint for each treatment, with the exception of WD at T1. This aligned with the ES genotype responding on a gene expression level to soil-drying at T1 while LS was not, while also highlighting the similarity of response between genotypes. Across all timepoints and water treatment states, there were 42 consistent DEGs between the ES and LS genotypes (21 upregulated and 21 downregulated in ES compared to LS), these included *A07G188640* a predicted cold and drought-regulated CORA-like protein (lower expression in ES than LS; Fig. 4b), *D09G007600* a terpene synthase 10-like (higher expression in ES than LS; Fig. 4c), and *D09G008150* a predicted transcription factor TCP13-like (higher expression in ES than LS) (Fig. 4d). Cold and drought-regulated CORA-like proteins and TCP13-like transcription factors are both implicated in drought responses.

### Metabolic characteristics of progressive soil-drying treatment

To build an understanding of the metabolic effects of the progressive soil drying treatment a combination of targeted and untargeted metabolomics was used to produce three non-mutually exclusive datasets (central carbon metabolism, Quadrupole Time-of-Flight Mass Spectrometry (QToF) in positive ion mode and QToF in negative ion mode). Metabolites were consolidated to form one dataset which contained peak intensity data for 494 unique metabolites. Of these, 410 had KEGG compound ID matches and these were used as background for over-representation analysis (ORA) for pathway enrichment results (Additional File 5).

**Fig. 5:**
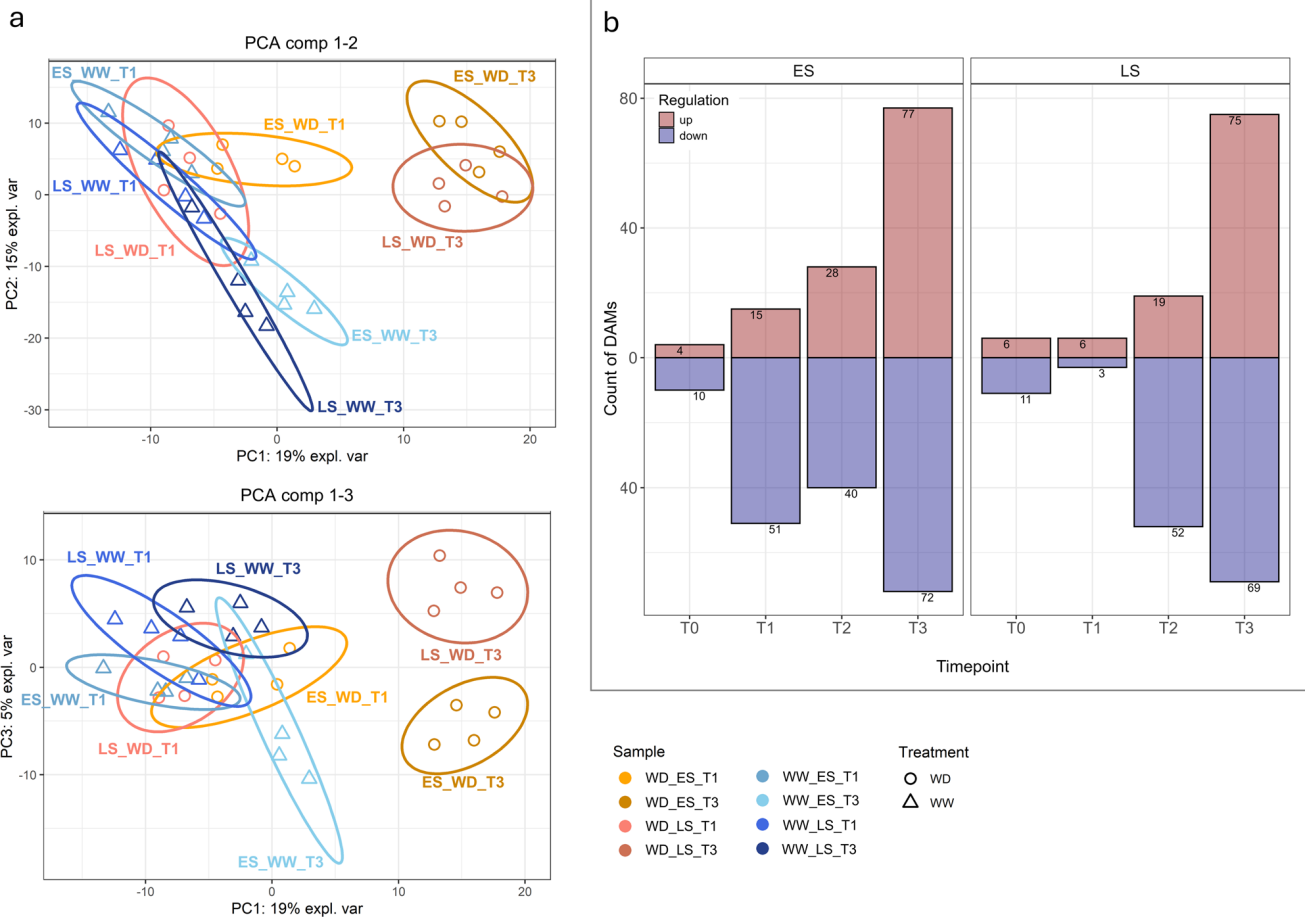
Leaf metabolic profiles of early-saver (ES) and late-saver (LS) genotypes and differentially expressed gene (DEG) gene counts between well-watered controls (WW) and water-deficit (WD) treated plants. **a** Principal component analysis of metabolome of ES and LS genotypes at T1 and T3 timepoints during progressive soil drying treatment. WW in blue shades and WD samples in orange shades. **b** DEG counts in ES and LS genotypes, between WD treated leaves and control samples

Principal component analysis (PCA) of samples found the first principal component explained 19% of the total variance, while PC2 explained 15% and PC3 a further 5%. The scores plots (Fig. 5a) showed PC1 clearly separated the samples by water-deficit treatment. Interestingly, PC2 separated the WW samples by timepoint, suggesting it was driven by metabolites linked to age/developmental state of the plants. While PC3 seemed to separate the two genotypes at most timepoints (Fig. 5a).

To find the metabolites responsive to the progressive soil drying treatment in each genotype, fold-change analysis with T-tests between WD-treated and control plants at each timepoint were performed. The differentially abundant metabolites (DAMs) counts mirrored that of the physiology data and DEG counts; increased with progression of the water-deficit treatment. A clear difference between genotypes at T1 was seen where the ES genotype had 66 DAMs while 9 were found in LS - a similar number to LS’s T0 secondary control of 17. While at T2, both genotypes had substantial metabolites impacted by the water-deficit treatment (68 in ES and 71 in LS). At T3, this had increased to 149 in ES and 144 in LS (Fig. 5b).

Only three DAMs were common between ES and LS at all timepoints when significant physiological changes were occurring due to soil-drying (T1, T2 and T3 in ES, and T2 and T3 in LS). These were 3-dehydroshikimic acid, pyridoxine and nicotinic acid.

Enrichment analysis of the KEGG pathways in the DAMs present in either genotype at any sampling timepoint showed no significant over-representation (raw *P* < 0.05) when using the full metabolome of 410 KEGG annotated metabolites as the background. To investigate further the underlying trends of DAMs in KEGG pathways, when the number of DAMs (which were part of a KEGG pathway) was greater than the pathway enrichment analyses’ expected number than that would be found by chance, the pathway was considered ‘of interest’ at that timepoint. For the list of these pathway enrichment ORA results see Additional File 6. At the T1 timepoint in ES, the pathways ‘glyoxylate and dicarboxylate metabolism’, ‘zeatin biosynthesis’, ‘pyrimidine metabolism’ and ‘ascorbate and aldarate metabolism were of interest. By T2, pathways of interest included ‘zeatin biosynthesis’, ‘aminoacyl-tRNA biosynthesis’ and ‘arginine biosynthesis’. At T3 there was ‘pentose and glucuronate interconversions’, ‘inositol phosphate metabolism’ and ‘galactose metabolism’. In the LS genotype, at T1, ‘glutathione metabolism' and 'ascorbate and aldarate metabolism’ were of interest with one metabolite hit each. At T2, of interest was ‘lysine degradation' and 'phenylalanine metabolism’ which had two metabolite hits each. By T3 the lowest p-value hits were ‘beta-alanine metabolism’, ‘carbon fixation in photosynthetic organisms’, ‘lysine degradation’, ‘pentose and glucuronate interconversions’, showing some similarity to the ES genotype’s T3 pathways.

**Fig. 6:**
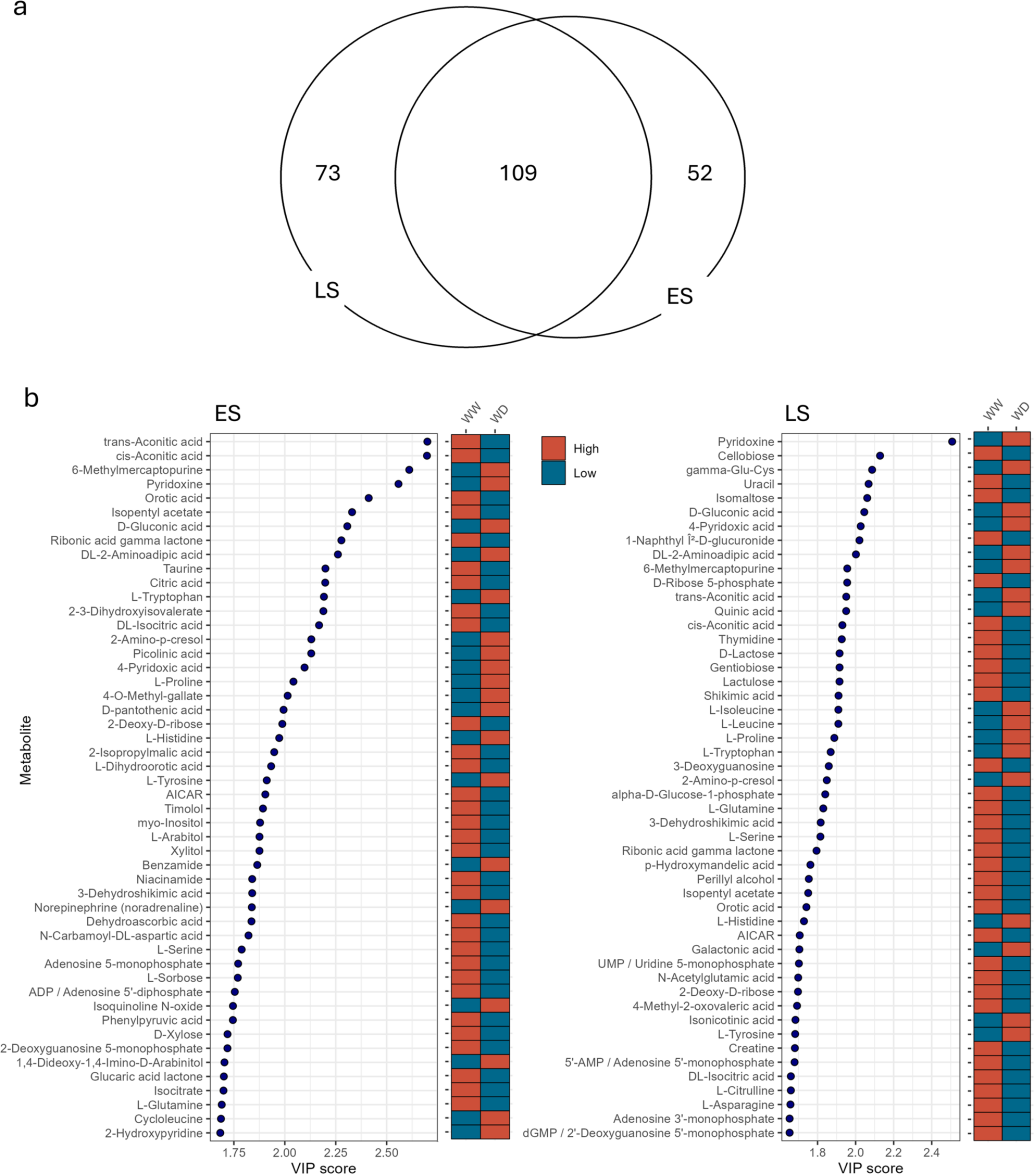
Partial Least Squares Discriminant Analysis (PLS-DA) results of leaf metabolome of each early-saver (ES) and late-saver (LS) genotypes during progressive soil drying treatment and controls. **a** Venn diagram of variable importance in projection (VIP > 1) metabolites between ES and LS genotypes during progressive soil drying treatment. **b** Top 50 metabolites with a VIP score > 1.0 from ES and LS genotypes during exposure to progressive soil drying. Coloured boxes on right indicate comparative relative abundance of metabolite in each condition

To investigate metabolites important during the progressive soil drying treatment, a sparse Partial Least Squares Discriminant Analysis (sPLS-DA) was performed for each genotype (all T0 samples were classified as “WW”), and high Variable Importance in the Projection (VIP) scores (cutoff > 1) metabolites identified. PLS-DA component 1 explained 14% of the total variation between control and progressive soil drying treatment in both genotypes. A pattern of increasing separation from T1 through to T3 in WD samples in both genotypes was evident and was more pronounced in ES than LS (Additional File 7, Fig.S1). The ES had 161 metabolites contributing importantly to the variation between control and WD, while LS had 182 metabolites. Both genotypes had 109 VIP metabolites in common where those that were DAMs at more than one timepoint responded in the same way (i.e. increased or decreased) (Fig. 6a). Among the top 25 VIP metabolites were L-Proline, trans-Aconitic acid, cis-Aconitic acid, 6-Methoylmercaptopurine, Pyridoxine, Orotic acid, Isopentyl acetate, D-Gluconic acid, Ribonic acid gamma lactone, DL-2-Aminoadipic acid, Taurine and Citric acid (Fig. 6b). Metabolites which accumulated during the progressive soil drying (WD treatment) were amino acids (such as L-Proline, L-Tryptophan, L-Tyrosine, L-Leucine and gamma-Aminobutyric acid (GABA)) and organic acids (such as D-Gluconic acid, DL-2-Aminoadipic acid, Galactonic acid and Isonicotinic acid). While depleted metabolites during the soil drying treatment were organic acids (such as cis-Aconitic acid, Orotic acid, Citric acid, DL-Isocitric acid, Isocitrate and Shikimic acid), amino acids (such as Taurine, Serine, Glutamine and Asparagine) and nucleotides and nucleosides such as (AMP, ADP, dGMP, Uridine and Guanosine).

### Differences and similarities in metabolite content between ES and LS

To investigate differences between metabolites affected in ES and LS during the progressive soil drying, an UpSet plot was used (Conway et al., 2017) (Additional File 7, Fig.S2). ES had 30 DAMs which were present at T1 which were not found at any point in the soil-drying treatment in LS. The top five of KEGG pathways for these were ‘Glyoxylate and dicarboxylate metabolism’, Pyrimidine metabolism’, ‘Zeatin biosynthesis’, ‘Ascorbate and aldarate metabolism’ and ‘Citrate cycle (TCA cycle)’. The three metabolites were part of the ‘glyoxylate and dicarboxylate metabolism’ pathway (linked to the tricarboxylic acid (TCA) Cycle) were: isocitrate with a 5.7-fold decrease; citrate with a 3.2-fold decrease; and L-glutamine with a 2.8-fold decrease.

To further investigate differences in DAMs between the two genotypes the sPLS-DA high VIP scores (cutoff > 1) metabolites were compared. There were 52 VIP metabolites associated only with ES and 73 only with LS (Additional File 8). Unique to ES included organic acids (such as Nicotinic acid, Pyruvic acid, β-Hydroxypyruvic acid, Glyceric acid, and Picolinic acid), amino acids (such as L-Aspartic acid, L-Homoserine and L-Phenylalanine), nucleotides and nucleosides (such as dUPT, dTP, AMP, XMP and CMP) and carbohydrates (such as D-Fructose 1,6-bisphosphate, D-Ribulose 5-phosphate and L-Sorbose), as well as Dehydroascorbic acid, the oxidised form of Ascorbic acid. While unique VIPs to LS included carbohydrates (such as Cellobiose, Isomaltose, Maltotriose and D-Glucose-1-phosphate), organic acids (such as Quinic acid, 4-Hydroxybenzoic acid, L-Hydroxyglutaric acid and Adipic acid), amino acids (such as L-Asparagine and L-Arginine) and nucleotides and nucleosides (such as dGDP, UMP, UDP-galactose, UDP-glucose, D-Ribulose 1,5-biphosphate) and phenolic compounds (such as Chyrsin, 5-HIAA and Luteolin).

**Fig. 7:**
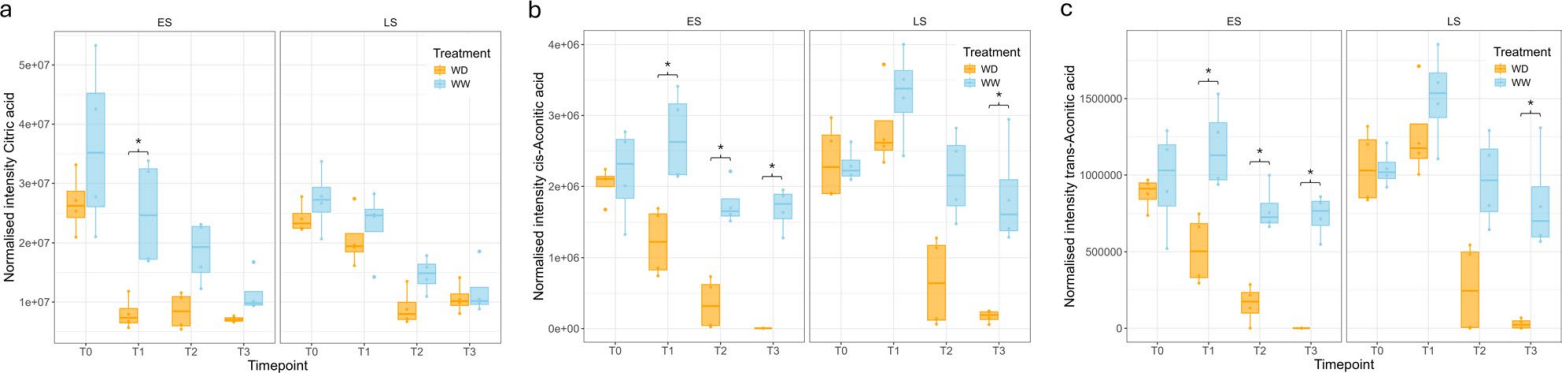
TCA cycle metabolites relative abundances in early-saver (ES) and late-saver (LS) genotypes during progressive soil drying treatment (WD) and controls (WW). A decrease can be seen in **a** Citric acid, **b** cis-Aconitic acid, and **c** trans-Aconitic acid in WD treated plants from T1 in ES, and from T2 in LS. Asterix above the WD and WW boxplots indicates a statistically significant difference between the treatments (Student’s t-test with *p* < 0.05 and |log2FC| > 0.5)

Noteworthy were the metabolites with the highest VIP scores in ES, trans- and cis-Aconitic acid. The relative abundances of these as well as other TCA cycle metabolite Citric acid were plotted (Fig. 7) showing a distinct reduction compared to controls in all three metabolites at the T1, T2 and T3 timepoint in ES, while this occurred at T2 and T3 in LS.

### Combined omics signatures of progressive soil drying treatment

To explore the relationship between transcriptome and metabolome in each genotype during the progressive soil drying treatment, a supervised multivariate sparse Partial Least Squares (sPLS) method was used [32]. Each genotype was studied separately in the sPLS analysis, to discover subsets of variables that are highly correlated in the data of each genotype, potentially highlighting any differences. The sPLS was performed using only the WD treated samples from T0 through to T3 in canonical mode, wherein the analysis treated both metabolites and transcripts symmetrically.

**Fig. 8:**
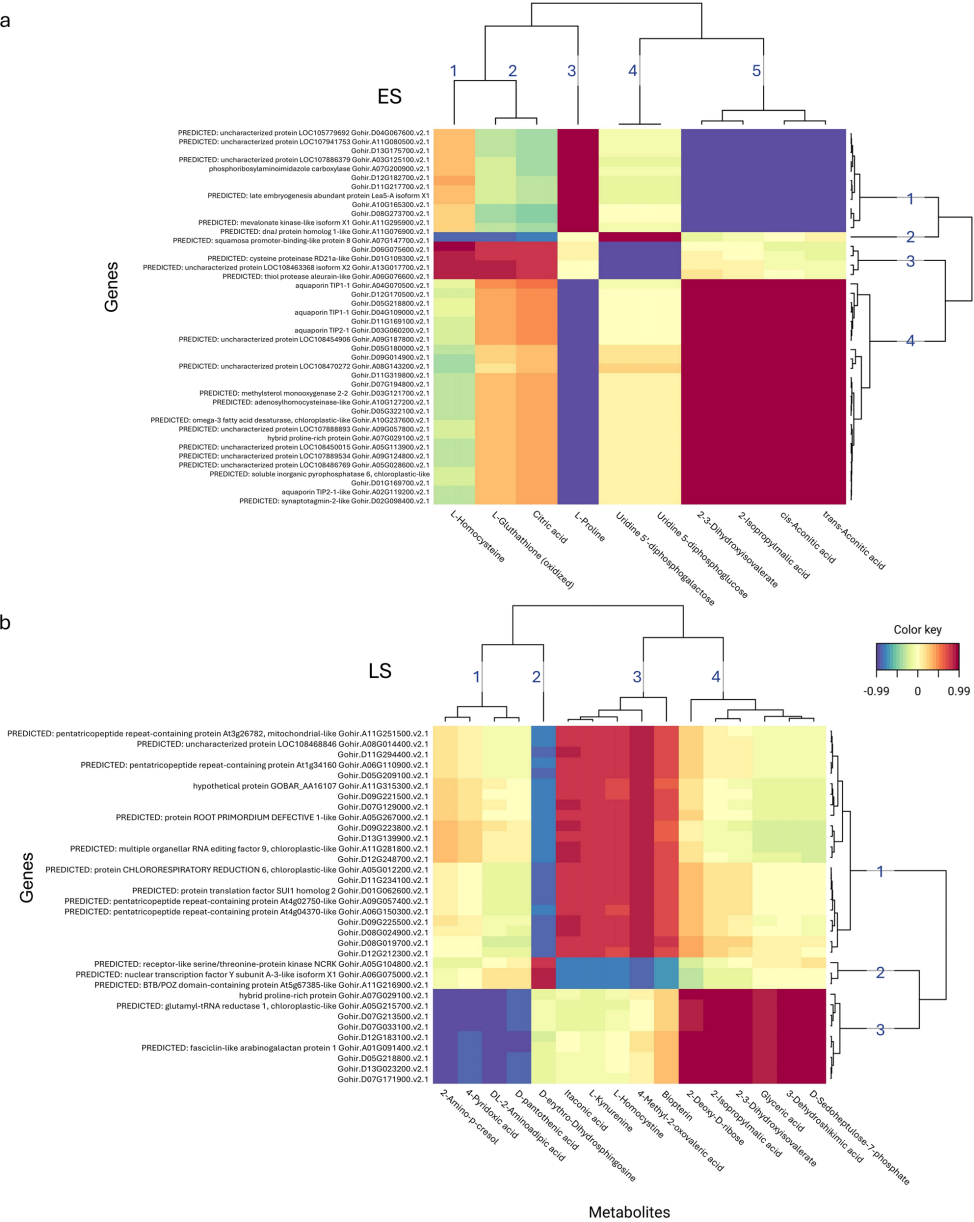
Clustered Image Maps from sPLS analysis of progressive soil drying (WD) treatment transcriptome and metabolome. The plot displays the similarity values between transcriptome and metabolome variables selected across two dimensions, and clustered with a complete Euclidean distance method. **a** Early-saver (ES), showing five clusters of metabolites and their associations to four clusters of genes. **b** Later-saver (LS), showing four clusters of metabolites and their associations to three clusters of genes.

The analysis revealed ES had five clusters of metabolites strongly associated with four clusters of genes (Fig. 8a). Genes from cluster 1 (see figure) which included phosphoiboxylaminoimidazole carboxylase and a predicticed Lea5-A gene, were strongly negatively associated with metabolites from cluster 5 which was made up of both cis- and trans-Aconitic acid, 2-Isopropylmalic acid and 2-3-Dihydroxyisovalerate. These same metabolites were then positively associated with a large cluster of genes (cluster 4) which notably included four aquaporin genes (two TIP1-1, a TIP2-1 and a TIP2-1 like). L-Proline formed its own metabolite cluster (cluster 3) which was negatively associated with these genes. This indicated when L-Proline accumulated and Aconitic acid decreased, then certain aquaporin transcript levels decrease simultaneously. Interestingly, a predicted transcription factor gene - squamosa promoter-binding-like protein 8 – showed distinct association to a few metabolite clusters (clusters 1, 2 and 4) which was the opposite to that of another cluster of genes (cluster 3), potentially indicating a regulatory role.

The LS analysis results produced four metabolite clusters with associations to three gene clusters (Fig. 8b). Of interest was gene cluster 2 which contained three predicted regulatory genes; a nuclear transcription factor Y subunit A-3-like gene; a receptor-like serine/threonine-protein kinase NCRK gene, and; a BTB/POZ domain-containing protein gene, which were positively linked to D-erythro-Dihydrosphingosine, a sphinogolipid, and negatively associated to metabolite cluster 3 which included L-Homocysteine and Itaconic acid. Metabolite clusters 1 (including 4-Pyridoxic acid and D-pantothenic acid) and 4 (including 3-Dehydroshikimic acid and D-Sedoheptulose-7-phosphate) were strongly associated with gene cluster 3. This included a hydrid proline-rich protein and three predicted pentatricopeptide repeat-containing protein genes (RNA-binding proteins potentially involved in post-transcriptional regulation of chloroplast genes).

## Discussion

Understanding the transcript and metabolic response to soil drying is crucial for developing cotton germplasm able to sense declining soil water availability early and respond accordingly. To investigate the differential physiological response of two closely related *G. hirsutum* genotypes displaying differing fraction of transpirable soil water (FTSW) thresholds (the soil water content at which a clear reduction in plant transpiration occurs), the leaf transcriptome and metabolome were analysed during a progressive soil drying treatment. Both the transcriptome and metabolome data were consistent with the early-saver (ES) genotype responding to the progressive soil drying treatment at a higher FTSW than late-saver (LS) (FTSW of approx. 25% at the T1 sampling) where the ES genotype showed a clear change in gene expression and metabolite abundances earlier (at T1) than LS (later, at T2), which was consistent with its higher FTSW threshold. After this point (T2 onwards), both genotypes had strong differential gene expression and metabolite abundance changes, indicating a systemic response to soil drying and water deficiency had occurred in both genotypes.

Gene expression changes in ES and LS were overall reasonably similar, once a response had begun. This was found by the majority of DEGs and DAMs being in common between the two genotypes. Pathways induced from the progressive soil drying were typical of a water-deprived state in both genotypes, including the downregulation of photosynthetic protein-coding genes, chlorophyll precursors (e.g. porphyrins) and carbon metabolism [34–36].

It is known cotton responds to water deprivation firstly by slowing its growth (through falling leaf water potential), followed by a suppression of photosynthetic activity [37]. The data show growth and photosynthesis were both impacted at the time of sampling for both genotypes. This was also seen in physiology data where the photosynthetic rate was reduced in ES by 10 days (T1) and LS by 14 days (T2) [17]. This also suggests that any potential differences in ‘sensing’ of the soil moisture decline by either genotype were likely to have occurred preceding the sampling timing of this experiment because photosynthetic rate was already impacted. Therefore, the noted differences in transcription between the genotypes after their response had begun suggests the closely related genotypes did differ slightly in their response to the progressive soil drying, rather than simply by their timing of sensing of soil drying. As any transcription changes involved in sensing of soil drying were potentially missed at the time of sampling, this is an area which could be addressed by a careful time course of sample collection to catch when growth is reduced before stomatal conductance and photosynthetic rate are suppressed.

Of particular interest were gene expression and metabolite changes present at the T1 timepoint that were unique to the ES genotype (not present in LS at any timepoint), or particularly pronounced at T1, as these potentially represented characteristics linked to its higher FTSW threshold trait. Two processes which were represented in the data were notably starch degradation product export from the chloroplast, due to unique gene expression of *MEX1* and *pGlct-2* found in ES at T1, and metabolites of the TCA cycle. There were also transcription factors with differential expression unique to each genotype, as well as genes which were consistently differentially expressed between genotypes regardless of treatment, such as a predicted cold and drought-regulated CORA-like protein gene.

### Starch breakdown genes unique to higher FTSW threshold genotype ES

Two starch metabolism protein coding genes were uniquely differentially expressed in the ES genotype and were differentially expressed at the T1 timepoint suggesting they were an early adaptation of ES to the progressive soil drying treatment. MEX1 and pGlct-2 each sit on the chloroplast envelope membrane and export maltose and glucose from the chloroplast, respectively [38–41]. These starch breakdown products are vital for providing energy for the plant from stored carbon (for example, during diel starch turnover, i.e. overnight breakdown). Mutants in *mex1* accumulate starch and maltose and are stunted in growth [40]. While the double mutant *mex1/pGlct-2* has a more severely stunted phenotype [41]. In the ES genotype, both transporters were upregulated at T1, if this translated to protein activity, it may potentially suggest an increase in starch breakdown. It is noted one particular MEX1 gene in ES, *A11G226000*, was consistently upregulated from T1 to T3 without any change in expression in LS, while an additional MEX1 gene (*D08G239900*) was upregulated in ES at T3 as well as LS at T3.

Generally, starch declines during stress such as drought, due to a reduction in synthesis and increase in breakdown (recently reviewed by [42]). In a non-stressed state, starch functions to non-osmotically store excess carbon during the day in the cell in plastid or chloroplast which is broken down to support plant growth and respiration overnight. During drought, it may be additionally broken down to provide carbon as sucrose or hexoses, and proline, which can function to sustain cellular processes and osmotically draw in water to cells for turgor maintenance. In both genotypes in this study, the transcript for the enzyme granule-bound starch synthase (GBSS) was upregulated from the earliest sign of response to soil drying (T1 in ES, T2 in LS). This enzyme converts ADP-G to amylose (starch), part of starch biosynthesis. While the starch-degrading enzymes alpha-amylose (AMY) and beta-amylase (BAM) genes were upregulated later (T2 in ES, T3 in LS), with the exception of one AMY transcript which was already upregulated in T2 in LS (*D12G170000*). This early upregulation of a GBSS gene could suggest an accumulation of starch had occurred in the early response to soil drying in both genotypes, while a greater breakdown of starch stores was initiated later. This is consistent with studies showing some plants can accumulate starch in response to the early stages of stress, for example, Hummel et al. [43] show during mild drought stress Arabidopsis accumulates starch due to rosette relative expansion rate reducing more than photosynthesis, temporarily producing a more positive carbon balance. Indicating starch may accumulate because growth is inhibited, but photosynthesis is not proportionately affected, yet [42, 44]. This is also consistent with cotton’s known initial drought response of slowing growth preceding a reduction in photosynthesis [37]. However, at the T1 sampling timepoint, photosynthesis was already suppressed in ES [17], implying a negative carbon balance and starch breakdown would predominate.

Further, an additional enzyme involved in amylase breakdown, isoamylase, was downregulated consistently in both genotypes as an early response (T1 in ES, T2 in LS). This may indicate a spatial difference in the leaf for starch accumulation and breakdown during the soil-drying response. It is possible ES had a differing approach in starch breakdown product export from chloroplasts of specific cell types such as guard cells or mesophyll cells. Performing starch assays on leaf tissues would help to clarify, as would additionally characterising the MEX1 and pGlct-2 genes and their protein’s localisations in the leaves of *G. hirsutum*.

This starch pathway is involved in the difference in response timing of the genotypes in the progressive soil drying treatment and warrants further investigation. A study by Dai et al. [45] found starch synthase (SS) to be involved in drought stress tolerance in cotton. As cotton is not grown for grain starch accumulation, unlike other crops, this pathway is found to be less well characterised in the literature than in other crops. Possible mechanisms through which starch may drive FTSW threshold differences in cotton could involve leaf starch storage variation (e.g. amount, branched and non-branched types, transiency, cell location, cell type, variation between leaf ages). Genotypes with a higher FTSW threshold may have greater innate starch reserves which are able to be used as a longer-term buffer, allowing for a longer period of reduced transpiration to ration water use while waiting for another rainfall. This could fit the early and sustained upregulation of genes involved in starch breakdown product export from chloroplasts seen in ES in this study (*MEX1* and *pGlct-2*). Further, starch breakdown-derived carbon allocation to root growth once the FTSW threshold has been passed may differ in higher and lower FTSW threshold genotypes and presents as an area of interest for future work.

### TCA / krebs cycle

The tricarboxylic acid (TCA) cycle, also known as the citric acid cycle or Krebs cycle, plays a fundamental role in cellular metabolism by oxidizing acetyl-CoA, derived from various carbon sources, to generate energy in the form of ATP and reducing equivalents and provides intermediates that serve as precursors for the biosynthesis of amino acids, lipids, and other essential molecules required for cell growth and function. During stress, such as drought, the TCA cycle plays an important role in stress responses by providing energy to compensate for a suppression of photosynthesis, to maintain redox balance, and synthesize stress-related compounds. For example, supplying reducing equivalents for antioxidant systems which scavenge ROS and protect cells from oxidative stress. Further, the intermediates of the TCA cycle serve as precursors for the biosynthesis of stress-related compounds, including amino acids, organic acids, and secondary metabolites such as phenolics. In ES, the two metabolites considered of greatest interest in the WD treatment were cis- and trans-aconitic acid (tAA), an intermediate between isocitrate and citrate which is central to the TCA cycle. The cis isomer is unstable and rapidly converted to isocitrate and functions within the TCA cycle. While tAA is considered to function outside of primary metabolism, and recently reported to be linked to drought tolerance in cacao [46]. Further, [47] shows tAA inhibits growth and photosynthesis in soybean when applied as a solution to the root system. tAA abundance was reduced in the leaves of both genotypes after responding to the progressive soil drying treatment (similarly with cis-aconitic acid). This may suggest a reduction in TCA cycle activity and a decrease of intermediates as a result. Citric acid, another important metabolite of the TCA cycle had decreased abundance in both WW and WD treated plants throughout the soil drying treatment, however it was strongly reduced in the ES WD treated plants. This additionally suggests a reduction in TCA cycle activity occurred, particularly in ES, during water deficiency. There was no change in expression of genes important in the TCA cycle including aconitate hydratase, aconitate isomerase, citrate synthase or citrate dehydrase at the ES T1 timepoint indicating the metabolic changes were not reflected on a gene expression level and could be the result of other regulatory mechanisms such as post-transcriptional regulation or protein activity changes.

### Transcription factors and other genes of interest

Transcription factors are of interest when searching for differences between genotypes due to playing a pivotal role in regulating gene expression and coordinating responses to environmental stresses, such as during drought. They control the expression of genes involved in related pathways such as signal transduction, and the synthesis of protective molecules, for example, osmoprotectants and antioxidants. In this study there were predicted transcription factors uniquely expressed early in the progressive soil drying response of ES and not seen to be differentially expressed in LS at this early stage, or at all. These were *A09G027100* a predicted myb-related protein 305 (downregulated in ES only), *D13G007900* a WRKY transcription factor 54 (downregulated in ES only), and *A12G175190* a predicted homeobox-leucine zipper protein HAT5-like (upregulated in ES only) (Table 1).

HAT5 is a homeodomain leucine zipper (HD-Zip) transcription factor implicated to be involved in abiotic stress in other crops. For example, HAT5 overexpressed in tomato leads to enhanced drought tolerance though greater accumulation of osmotic regulatory substances (especially proline) and enhances antioxidant enzyme activities [48]. Other HD-Zip transcription factors are found to respond to stress in cotton such as GhHB12 which represses jasmonic acid related genes [49]. As the predicted HAT5 gene *A12G175190* was upregulated at T1 in ES, it is possible it was involved in regulating genes involved in the soil-drying response. This and other transcription factors uniquely differentially expressed in ES could be further investigated by overexpression or knockout mutant studies or predicting HAT5 regulated genes through promoter region motifs.

Another gene uniquely differentially expressed in ES from T1 (considered an early and consistent response) included *A05G048700* a BURP domain RD22-like precursor. BURP domain-containing genes, such as those in the RD22 subfamily, encode a group of plant proteins characterized by the presence of a unique BURP domain. The RD22 subfamily is known for its involvement in various physiological processes, including stress responses and can be induced by drought, abscisic acid (ABA), and salt stress [50]. Given this predicted RD22-like gene was upregulated only at the T1 timepoint, and only in ES, suggests it a) may have been a transient upregulation during the early stages of response to dehydration in ES (therefore not present at T2 or T3). If this was the case, it is possible this transient upregulation occurred in LS but was missed due to the timing of sampling. Or b), it is possible this gene is responsive to water-deficit in ES but not in LS. In this case further characterisation and expression studies would be of interest. BURP encoding genes in cotton have been identified by Sun et al. [51] where stress-responsive cis-regulatory elements are found in *BURP* promoters, with the RD22-like subfamily being induced by various stresses involving abscisic acid (ABA) as well as salicylic acid (SA). GhRD22 genes have also been associated with fiber growth and quality in cotton [52, 53].

A predicted cold and drought-regulated CORA-like protein gene *A07G188640* was of interest due to having a consistent, significantly lower expression level in the ES genotype, furthermore, its expression level remained similar to controls throughout the soil drying treatment while in LS the same gene responded to the treatment with significant upregulation. Therefore, *A07G188640* may have contributed to the lower FTSW threshold of LS, potentially providing a level of protection to growth in the early stages of the water deficit through higher expression levels. Jha et al. [54] introduced a CORA-like gene from an extreme haplotype into tobacco whose overexpression improved plant growth under drought stress. To investigate this further the abundance of the protein form is of interest, as well as studying the phenotypes of mutants.

### Correlated features in transcriptome and metabolome

Features that exhibit correlation between transcriptome and metabolome data provide information of the molecular interactions and regulatory mechanisms involved in the progressive soil drying treatment response.

Notable was the negative association in ES of four known aquaporin gene transcript levels (two *TIP1-1*, one *TIP2-1* and a *TIP2-1-like*) to the accumulation of L-Proline. Aquaporins play an important role in regulating water transport across membranes. The TIP group of aquaporins specifically are known to sit on the tonoplast membrane. A study by Guo et al. [55] silenced *GhTIP2-1* in cotton to produce plants with a greater sensitivity to osmotic stress, while overexpression led to reduced H_2_O_2_ and malondialdehyde content (indicators of oxidative stress) and higher proline content. Cheng et al. [56] show *GhTIP1-1* expression is induced by drought stress. This is in contrast to the effect of the progressive soil drying treatment used in this study, which led to downregulation of multiple aquaporin genes.

In LS, the same aquaporin transcripts, except for TIP2-1-like (*A02G119200*) were also downregulated in the progressive soil drying treatment (starting at T2), however L-Proline did not show marked accumulation at the same timepoint, only later at T3. This shows an interesting relationship between TIP aquaporin transcript levels, proline and water-deprivation response. This area may be exploited to produce potentially ‘more sensitive’ cotton plants leading to a greater level of water conservation for growth, ideal for rainfed production systems. Further studies to clarify the effect of the different aquaporin genes, how expression relates to protein levels, and their effect on drought sensitivity is required.

## Conclusions

**Fig. 9:**
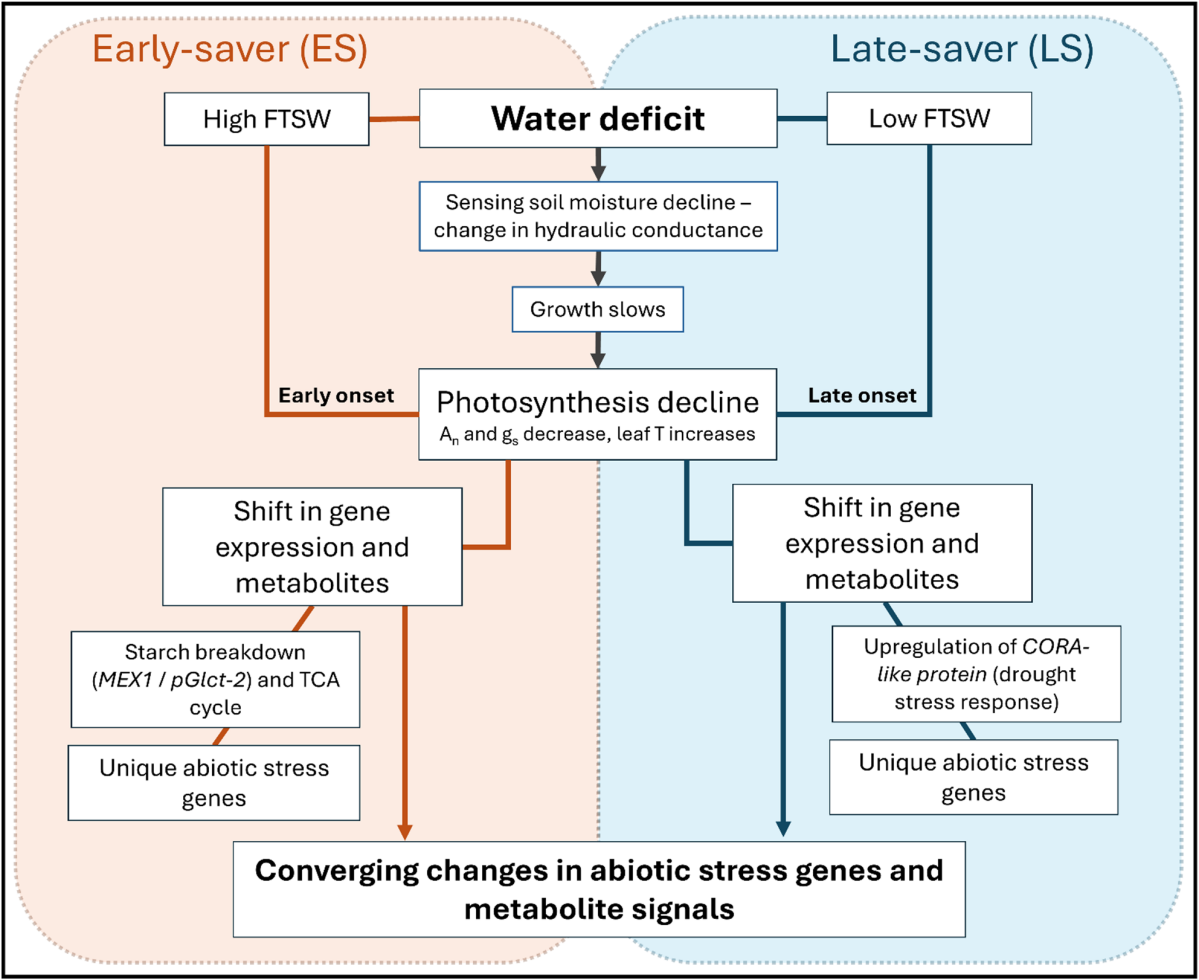
A graphical representation of early-saver (ES) and late-saver (LS) genotype’s response to declining soil moisture encompassing physiology data collected by Broughton et al. [17] and gene and metabolite data collected. Both genotypes slowed down photosynthesis in response to reaching their FTSW thresholds and subsequently had some differing regulation of genes and metabolites. Abbreviations: A_n_ (photosynthetic rate); g_s_ (gas exchange); and leaf T (leaf transpiration).

This study used transcriptome and metabolome data to reveal metabolism patterns in two closely related *G. hirsutum* genotypes experiencing progressive soil drying. We hypothesised that molecular profiles would be similar between the two genotypes, however the timing and scale of each genotype’s response would differ, matching physiology data [17]. Metabolome and transcriptome data matched the physiological response in both genotypes, with substantial expression changes present 10d (T1) into the progressive soil drying treatment in the early-saver (ES) genotype (which had reached its fraction of transpirable soil water (FTSW) threshold), while, at the same timepoint, the late-saver (LS) genotype had not responded. However, the genotypes showed differences in response beyond that of timing (Fig. 9). We have shown the ES genotype had unique gene expression during the soil-drying notably in starch breakdown product export from the chloroplast and a more prominent early effect on the TCA cycle (Fig. 9). Integration of metabolome and transcriptome showed associations between genes and metabolites in each genotype, notably multiple aquaporin genes with L-proline, suggesting these may be key players in the broad response of cotton to progressive soil-drying. Validating the functionality of the drought-responsive genes or metabolic pathways found to be of interest in this research will help to understand the mechanisms of drought tolerance at the molecular level, aiding in the development of cotton genotypes with improved productivity and adaptation to water limited and rainfed production systems. 

## Supplementary Information

Additional file 1. Table S1. Differentially expressed genes (DEGs) present in early-saver (ES) and late-saver genotypes at T1 (ES only), T2 and T3 sampling timepoints during progressive soil drying treatment.

Additional file 2. Table S2. GO term and KEGG pathways enriched in the common differentially expressed genes (DEGs) between early-saver (ES) and late-saver LS) genotypes during progressive soil drying treatment.

Additional file 3. Table S3. GO term and KEGG pathways uniquely enriched in early-saver (ES) and late-saver (LS) differentially expressed genes (DEGs) during progressive soil drying treatment.

Additional file 4. Table S4. Genes that were consistently differentially expressed (present at each timepoint after FTSW threshold had been reached) that were unique to the late-saver (LS) genotype found using UpSet/Venn diagrams.

Additional file 5. Table S5. Metabolites and identification information which comprised the metabolome used in the study.

Additional file 6. Table S6. Pathway enrichment results of differentially abundant metabolites (DAMs) present in either early-saver (ES) or late-saver (LS) genotypes at T1, T2 and T3 sampling timepoints during progressive soil drying treatment. Pathway enrichment performed using over-representation analysis (ORA) of KEGG pathways.

Additional file 7. Figure S1. Partial Least Squares Discriminant Analysis (PLS-DA) component 1 and component 2 projection for metabolome of early-saver (ES) and late-saver (LS) genotypes to investigate metabolites contributing to difference between control leaf samples and those treated with progressive soil drying treatment. PLS-DA component 1 explained 14% of the total variation between control and progressive soil drying treatment in both genotypes. A pattern of increasing separation from T1 through to T3 in WD samples in both genotypes was evident and was more pronounced in ES than LS. Figure S2. UpSet plot to investigate differences and similarity of differentially abundance metabolites (DAMs) between early-saver (ES) and late-saver (LS) genotypes at different timepoints (T0, T1, T2 and T3) during the progressive soil drying treatment.

Additional file 8. Table S7. Metabolites with Variable Importance in the Projection (VIP) scores > 1 from sparse Partial Least Squares Discriminant Analysis (sPLS-DA) unique to either early-saver (ES) or late-saver (LS) genotype. There were 52 VIP metabolites associated only with ES and 73 only with LS.

## Data Availability

The RNA-seq dataset analysed during the current study is available in the NCBI Sequence Read Archive (SRA) repository under BioProject PRJNA1338341. The metabolome dataset is available upon request.
