# Supplementary material for: Transcriptome and metabolome insights into closely related upland cotton (Gossypium hirsutum) genotypes during differing responses to progressive soil drying
Source: BMC Plant Biol. 2026 Jan 29;26:367. doi: 10.1186/s12870-026-08125-5 (PMC12924551; doi:10.1186/s12870-026-08125-5)
Supplement: Supplementary file 7 — Additional file 7. Figure S1. Partial Least Squares Discriminant Analysis (PLS-DA) component 1 and component 2 projection for metabolome of early-saver (ES) and late-saver (LS) genotypes to investigate metabolites contributing to difference between control leaf samples and those treated with progressive soil drying treatment. PLS-DA component 1 explained 14% of the total variation between control and progressive soil drying treatment in both genotypes. A pattern of increasing separation from T1 through to T3 in WD samples in both genotypes was evident and was more pronounced in ES than LS. Figure S2. UpSet plot to investigate differences and similarity of differentially abundance metabolites (DAMs) between early-saver (ES) and late-saver (LS) genotypes at different timepoints (T0, T1, T2 and T3) during the progressive soil drying treatment. [file 12870_2026_8125_MOESM7_ESM.docx]

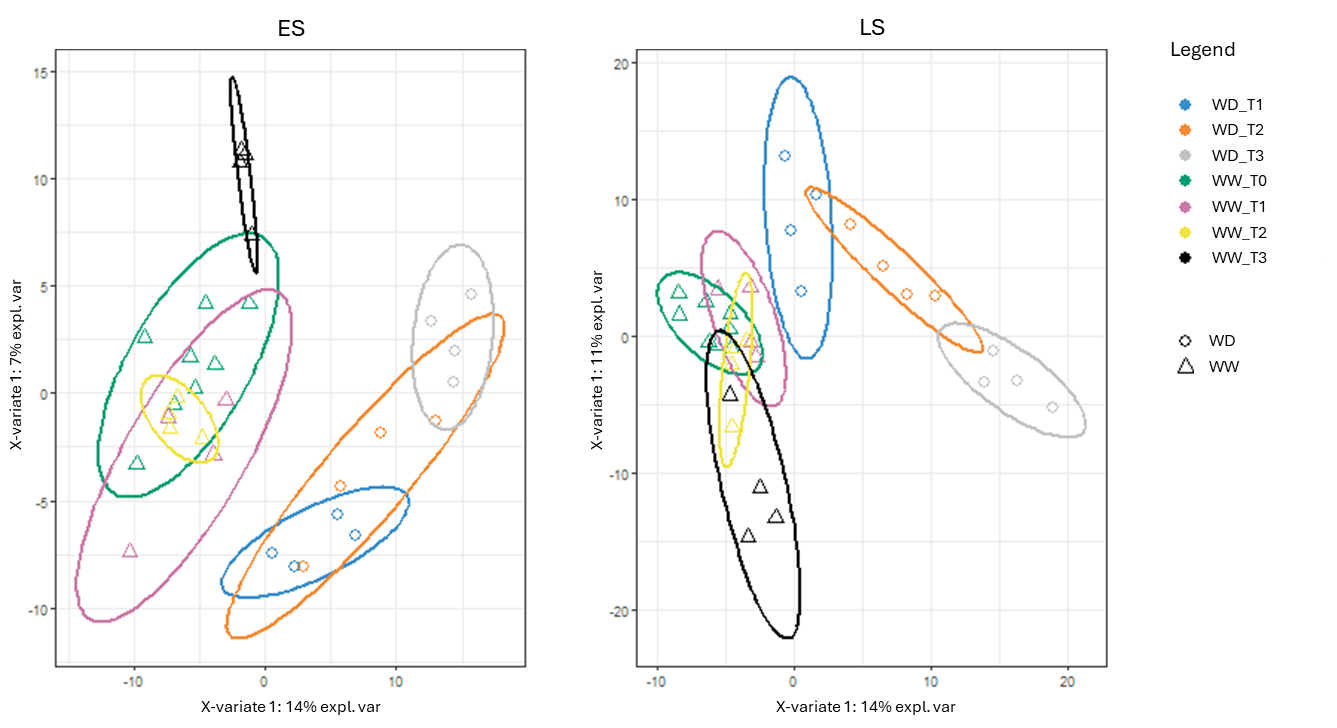


**Figure S1**. Partial Least Squares Discriminant Analysis (PLS-DA) component 1 and component 2 projection for metabolome of early-saver (ES) and late-saver (LS) genotypes to investigate metabolites contributing to difference between control leaf samples and those treated with progressive soil drying treatment. PLS-DA component 1 explained 14% of the total variation between control and progressive soil drying treatment in both genotypes. A pattern of increasing separation from T1 through to T3 in WD samples in both genotypes was evident and was more pronounced in ES than LS.


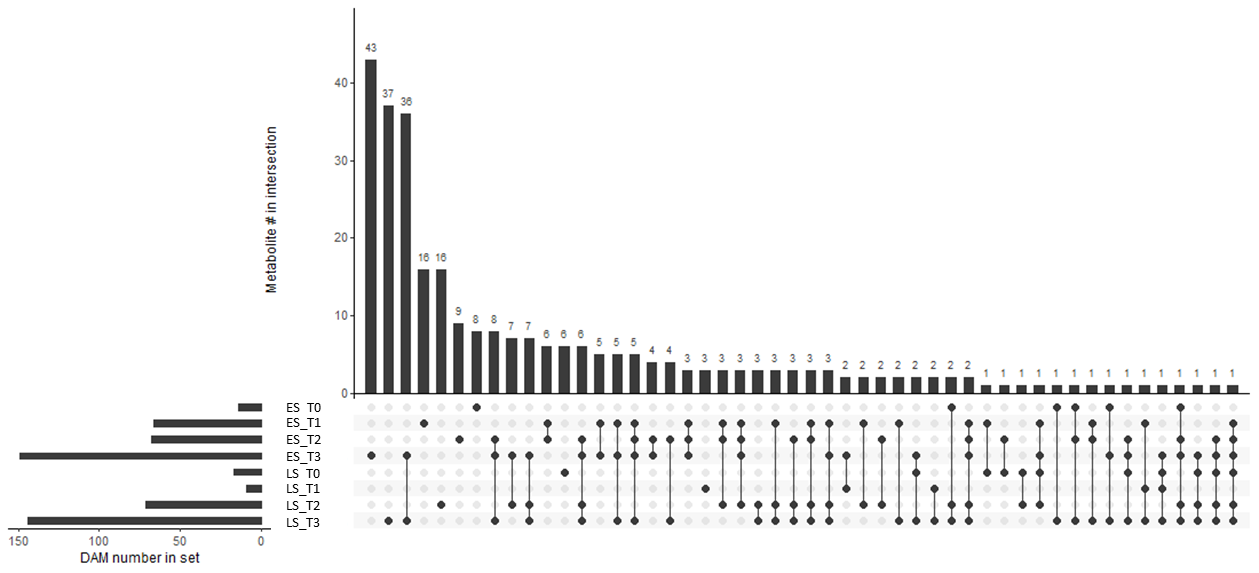


**Figure S2**. UpSet plot to investigate differences and similarity of differentially abundance metabolites (DAMs) between early-saver (ES) and late-saver (LS) genotypes at different timepoints (T0, T1, T2 and T3) during the progressive soil drying treatment.
